# Reward for Kindness

**Published:** 1858-12

**Authors:** 


### REWARD FOR KINDNESS.

BETWEEN a kindness to a fellow creature from the spontaneous welling up of a loving heart, and the serpent-like venom which a revengeful nature meanly spits out to injure his brother man, how immeasurable the distance; what infinite sweetness brings the former, and what an eating agony the latter, till life's latest hour, on every occasion, when in moments of retirement, the busy memory wakes up the scenes of the past. Besides, there is no reward for any deed of wrong that has in it one spark of gladsomeness, either now or hereafter; but for a good act, perennial springs of delicious memories run out and ripple along the side of all life's subsequent pathway, while unexpected rewards are now and then showered down into the very laps of the generous and the merciful.

On the battle field of Chalmette, a wounded soldier laid helpless on the ground, and almost expiring; his friend and comrade bore him away to a place of shelter, and nursed him into life again. Just fifty years later, Judah Truro left Shephard, his faithful friend, nine hundred thousand dollars.

A late advertisement reads:

"PERSONAL.—$45,000,"—"If Mr. JOSEPH ASHLEY, supposed to be a resident of New York, U. S., who in 1839, resided temporarily in Liverpool, and there befriended a young man named WILLIAM EMMERSON, will apply to WM. HARDMAN, 118 *Norton street*, *Liverpool*, he will hear of property to the above amount, bequeathed to him by said EMMERSON—recently deceased in Demarara.

In copying the above without charge, except to the credit of good-doing, the editor of the *Yonkers Herald* makes the following manly comment:

"We assure our readers that no paid advertisement has been inserted in our columns, with more pleasure than the above. It gives us reason to hope that Mr. JOSEPH C. ASHLEY, or his heirs, will receive the money left him by one he has befriended. We wish to add, what is a secret to a miser, that Mr. ASHLEY has received that which is more than both principal and interest for "befriending" Mr. EMMERSON, in the luxury that inherently accompanies the consciousness of doing our duty, in helping our fellow beings, especially such an one as Mr. EMMERSON, whose evidence of gratitude lives, when he is dead, and whose life-long desire was, to repay if possible, a man to whom he owed nothing, and who had only done his duty. Were we one of Mr. ASHLEY'S children, we would be pleased to receive the money; it would not, if well used, lessen the pleasure, but we cannot for a moment suppose it could please us more than would the legacy of his good name, and the knowledge of our being the son of a father whose kind actions are chasing him through the world after a lapse of nineteen years."

